# Development of Nine Markers and Characterization of the Microsatellite Loci in the Endangered *Gymnogobius isaza* (Gobiidae)

**DOI:** 10.3390/ijms13055700

**Published:** 2012-05-11

**Authors:** Kiwako S. Araki, Takefumi Nakazawa, Atsushi Kawakita, Hiroshi Kudoh, Noboru Okuda

**Affiliations:** Center for Ecological Research, Kyoto University, Hirano 2-509-3, Otsu 520-2113, Japan; E-Mails: take.nkzw@gmail.com (T.N.); kawakita@ecology.kyoto-u.ac.jp (A.K.); kudoh@ecology.kyoto-u.ac.jp (H.K.); nokuda@ecology.kyoto-u.ac.jp (N.O.)

**Keywords:** bottleneck, formalin-fixed samples, *Gymnogobius isaza*, Lake Biwa, microsatellite

## Abstract

*Gymnogobius isaza* is a freshwater goby endemic to Lake Biwa, Japan. They experienced a drastic demographic bottleneck in the 1950s and 1980s and slightly recovered thereafter, but the population size is still very small. To reveal dynamics of genetic diversity of *G. isaza*, we developed nine microsatellite markers based on the sequence data of a related goby *Chaenogobius annularis*. Nine SSR (Simple Sequence Repeats) markers were successfully amplified for raw and formalin-fixed fish samples. The number of alleles and expected heterozygosities ranged from one to 10 and from 0.06 to 0.84, respectively, for the current samples, while one to 12 and 0.09 to 0.83 for historical samples. The markers described here will be useful for investigating the genetic diversity and gene flow and for conservation of *G. isaza*.

## 1. Introduction

*Gymnogobius isaza* (Gobiidae) is a freshwater goby endemic to Lake Biwa, Japan. This goby is somewhat unusual in that it has been adapted to a pelagic habitat with its strong swimming ability, whereas most other gobiid fish are benthic. In addition, *G. isaza* plays important roles in the lake ecosystem as a keystone predator coupling pelagic and benthic food webs by diel vertical migration [1]; Briones *et al.*, unpublished data and also as a socioeconomically valuable fishery target. Fishery data [2] show that the population suddenly collapsed (<1%) in the 1950s and 1980s. The population decline in the 1980s is probably attributable to combined effects of various ecosystem disturbances including lakeshore habitat degradation [3], explosion of introduced piscivores [4,5], and warming-induced hypoxia in the deep water [6]. Although the population recovered slightly thereafter in the late 1990s, it is still relatively low and thus this goby is now categorized as “critically endangered (CR)” in the Red Data Book of Japan [7]. We expect that the genetic diversity of *G. isaza* has been substantially reduced due to the demographic bottleneck. This can exert strong negative impacts on individual fitness, population dynamics, and future extinction risk of the fish [8,9], and thus be crucial for its species conservation and resource management. At present, however, nothing is known about the genetic consequences of the demographic bottleneck because no information is available for its genetic components. Fortunately, specimens of *G. isaza* have been archived since 1962 by the Center for Ecological Research, Kyoto University, where the specimens were initially fixed in 10% formalin and subsequently preserved in 70% ethanol [10,11]. In this study, in order to reveal changes in genetic diversity from current and archived *G. isaza* samples, we developed microsatellite markers for *G. isaza* from sequences of related species.

## 2. Results and Discussion

With 15 primer pairs, nine loci were successfully amplified from both raw and formalin-fixed samples (Table 1). The number of alleles per locus ranged from one to 10 for 18 frozen current samples and one to 12 for 32 formalin-fixed historical samples (Table 2). For the current samples, the values of observed and expected heterozygosities ranged from 0.06 to 0.78 and 0.06 to 0.84, respectively, while 0.00 to 0.72 and 0.09 to 0.83 for the historical samples. Linkage disequilibria between pairs of loci and deviations from the Hardy-Weinberg equilibrium were also tested. *Isaz*5 significantly deviated from the Hardy-Weinberg equilibrium, suggesting null alleles in this locus. No significant linkage disequilibria were detected.

## 3. Experimental Section

We downloaded a data source of 15 DNA sequences containing microsatellite repeats (Accession No. AB557719 to AB557733) of *Chaenogobius annularis* (Gobiidae) from DDBJ (DNA Data Bank of Japan) [12]. Primer pairs were designed using Primer 3 (v.0.4.0) [13]. Simultaneously, to design primers at more conservative regions, homologous sequences in *Danio rerio* were searched by using BLASTN system in NIBB [14] and *C. annularis* sequences as queries. Conservative regions were identified by visual inspection, and additional primers were designed accordingly.

Total genomic DNA was extracted from fins by incubating with 600 μL of 1 × STE buffer, 60 μL of 10% SDS and 300 μg proteinase K at 55 °C for three days and then 300 μg proteinase K were added every 24 h. The sample was then extracted by Tris/phenol, phenol/chloroform/isoamyl alcohol and chloroform/isoamyl alcohol, in this order, before ethanol precipitation and washing. Although we used the above protocol to extract DNA for both frozen and formalin-fixed samples, our preliminary examination indicated that a simplified method with 3 h proteinase K incubation and without Tris/phenol and phenol/chloroform/isoamyl alcohol treatments sufficient for frozen (flesh) samples. PCR was performed with 5 ng of extracted DNA in final volume, 10 μL of 1 × Ex Taq HS buffer (TaKaRa, Otsu, Japan) and 10 μM of both forward and reverse primers. According to the manufacturer’s recommendation, amplification was done with initial denaturation of 3 min at 94 °C, followed by 45 cycles of 30 s at 94 °C, 30 s at annealing temp (56 or 58 °C), and 1 min at 72 °C, with a final extension for 5 min at 72 °C. The PCR product size was measured using an ABI PRISM 3130 Genetic Analyser with GeneMapper analysis software (Applied Biosystems, Carlsbad, California, USA). Nine primer pairs successfully yielded PCR products. To confirm that the amplified products contain the target microsatellite regions, these fragments were ligated to the pGEM-T easy vector in accordance with the manufacturer’s instruction (Promega, Madison, Wisconsin, USA). Recombinant clones were identified by using blue/white screening on Luria-Bertani agar plates containing ampicillin, X-Gal and IPTG. Insert-positive clones were amplified using M13 primers and sequenced using a BigDye Terminator Cycle Sequencing Kit (Applied Biosystems, USA). Complete sequences of all of nine loci including target microsatellite regions were determined as published in DDBJ (Accession No. AB703105 to AB703113).

To confirm effectiveness of designed primers for investigating genetic diversity, 18 frozen (current) and 32 formalin-fixed (historical) samples were used for the examination of polymorphisms for all 15 loci. Frozen samples were collected from the northwest shore of the Lake Biwa in 2010 and kept in −40 °C freezer. Formalin-fixed samples were caught in 2002 and fixed in formalin immediately. Polymerase chain reactions (PCRs) were performed with the standard protocol of the QIAGEN Multiplex PCR Kit (QIAGEN), in a final volume of 10 μL, which contained 0.2 μM of each multiplexed primer, 5 μL of Multiplex PCR Master Mix and 5ng of extracted DNA. Forward primers were labeled with the fluorochrom 6-FAM or VIC, NED or PET (Applied Biosystems, USA). The amplification profiles included initial denaturation at 95 °C for 15 min; followed by 30 (40 for formalin-fixed samples) cycles of 30 s at 94 °C, 1.5 min at appropriate annealing temperature, and 1 min at 72 °C; then final extension at 60 °C for 30 min. The primer pairs of *Isaz9*, *10*, *12*, and *15* were simultaneously amplified at 56 °C annealing temperature and *Isaz1*, *2*, *4*, *5* and *14* at 58 °C (see Table 1 for details). The observed and expected heterozygosities were calculated by using GENEPOP version 4.0.10 [15].

## 4. Conclusion

Developed SSR (Simple Sequence Repeats) markers described here will be used for both formalin-fixed and fresh frozen samples and useful for investigating genetic diversity and population dynamics in *G. isaza*. These markers developed for *G. isaza* are also potentially useful for assessing genetic diversity and gene flow of other *Gymnogobius* species.

## Figures and Tables

**Table 1:** Characteristics of nine microsatellite loci in *Gymnogobius isaza*. Shown for each primer pair are the forward (F) and reverse (R) sequence, repeat type, size range of the fragment, labeling dye, optimal annealing temperature (Ta) and accession number.

Locus	Primer Sequence (5′~3prime;)	Repeat	Size Range (bp)	Labeling dye	*T*_a_ (°C)	Accession No.
Isaz1	F: CTGAAGGTCAGAGGTCAGAGGTCA	(GT)_4_GAGGGGCGTGGCTAAAGCTA(GT)_4_	225–252	PET	58	AB703105
	R: GGGAAAGACATGAGGCAAAA					
Isaz2	F: GGAGAGGAGAAAGGGTTTGG	(AG)_10_	238–224	6-FAM	58	AB703106
	R: CAGGCTCCTTACCTCCAGTG					
Isaz4	F: CCATCACTCTCGCTCTGTT	(CT)_3_TT(CTCTCGTT)_2_ (CT)_3_TT(CT)_3_	180	NED	58	AB703107
	R: AGAGACAACCTGCCTTCAGA					
Isaz5	F: ATGAGGATGTCACAAGTGGAGCAG	(GA)_18_	168	6-FAM	58	AB703108
	R: CTGGGTGAATGTAGGGCAGT					
Isaz9	F: ATGGACAAGTCGGAAACTCG	(AC)_13_	150–165	VIC	56	AB703109
	R: AAAGTTTCTAAAGACCAACAC					
Isaz10	F: GGTTTGTCCCACTTTGCCTGT	(CA)_4_AA(CA)_2_TA(CA)_2_	144–151	NED	56	AB703110
	R: GTGAAAGGTTGTGCATGTGG					
Isaz12	F: CTTGGCATGAAATTGGCTTT	(GA)_14_	323–333	VIC	56	AB703111
	R: CCTGCTTTCTATTCCCAGTTGTAA					
Isaz14	F: ATCTGTATCGCCTCCTTTCTCC	(CT)_11_	125–162	VIC	58	AB703112
	R: GCGGTTCAAAGCCCCGGTCTGT					
Isaz15	F: CATAGCACCGCCTAGTGTGA	(CA)_8_	178–198	NED	56	AB703113
	R: ACTCCCACGGACGAATACTG					

**Table 2:** Results of nine microsatellite primers in two populations of frozen current and formalin-fixed historical samples of *Gymnogobi isaza*.

	Frozen Current (2010, *N* = 18)			Formalin-Fixed Historical (1983–2002, *N* = 32)		

Locus	*A*	*H*_o_	*H*_e_	*A*	*H*_o_	*H*_e_
Isaz1	8	0.56	0.75	8	0.72	0.77
Isaz2	2	0.06	0.06	3	0.09	0.09
Isaz4	1	n.s.	n.s.	1	n.s.	n.s.
Isaz5	1	n.s.	n.s.	4	0.00	0.62
Isaz9	10	0.33	0.29	8	0.59	0.74
Isaz10	2	0.78	0.84	4	0.22	0.47
Isaz12	6	0.72	0.76	7	0.56	0.80
Isaz14	7	0.72	0.83	9	0.63	0.83
Isaz15	7	0.50	0.61	12	0.22	0.56

*N*: number of individuals; *A*: number of alleles; *H*_o_: observed heterozygosity; *H*_e_: expected heterozygosity; n.s.: not stated because of *A* = 1.
